# Possibilities of Preoperative Medical Models Made by 3D Printing or Additive Manufacturing

**DOI:** 10.1155/2016/6191526

**Published:** 2016-06-28

**Authors:** Mika Salmi

**Affiliations:** School of Engineering, Department of Mechanical Engineering, Aalto University, Otakaari 4, 02150 Espoo, Finland

## Abstract

Most of the 3D printing applications of preoperative models have been focused on dental and craniomaxillofacial area. The purpose of this paper is to demonstrate the possibilities in other application areas and give examples of the current possibilities. The approach was to communicate with the surgeons with different fields about their needs related preoperative models and try to produce preoperative models that satisfy those needs. Ten different kinds of examples of possibilities were selected to be shown in this paper and aspects related imaging, 3D model reconstruction, 3D modeling, and 3D printing were presented. Examples were heart, ankle, backbone, knee, and pelvis with different processes and materials. Software types required were Osirix, 3Data Expert, and Rhinoceros. Different 3D printing processes were binder jetting and material extrusion. This paper presents a wide range of possibilities related to 3D printing of preoperative models. Surgeons should be aware of the new possibilities and in most cases help from mechanical engineering side is needed.

## 1. Introduction

3D printing is a process where material is added usually layer by layer as opposite to traditional manufacturing methods. In medical field, 3D printing has huge potential since every patient is unique and customization with 3D printing required only modifying the 3D model of the product [1].

Medical applications of 3D printing (additive manufacturing) can be categorized into five different groups: (1) medical models, (2) medical aids, orthoses, splints, and prostheses, (3) tools, instruments, and parts for medical devices, (4) inert implants, and (5) biomanufacturing [2]. Similar older application based classifications can be also found by different researchers [3–6]. Hopkinson et al. [3] used terms presurgery rapid manufacturing, orthodontics, drug delivery devices, limb prosthesis, and in vivo devices. Otherwise, Gibson et al. [4] called them surgical and diagnostic aids, prosthetics, and organ printing. In 2009 Mäkitie et al. classified in preoperative planning, surgical training, and teaching, inert implants, surgical instruments, and special equipment associated with the operations, postoperative guides, long-term supports and aids, and artificial tissue. In 2010 Tuomi et al. divided applications to models for preoperative planning, education, and training, inert implants, tools, instruments, and parts for medical devices, medical aids, supportive guides, splints and prostheses, and biomanufacturing. Examples of 3D printing applications include dental applications such as bite splints [7], hard and soft oral applications [8, 9] and trachea scaffolds [10], and surgical instruments made by 3D printing [11].

Scientific research has highly focused on implants and biomanufacturing but recently more and more medical preoperative models are made by using 3D printing for patient treatment. There are multiple cases related to craniomaxillofacial and dental surgery [12–14] and even lots of researches related to accuracy of the medical models made by 3D printing [15, 16]. Also guidelines for medical imaging related to making medical models by 3D printing can be found [17], where one major parameter is layer thickness of imaging.

The accuracy of binder jetting has been found varying from 0.69% to 0.38% [18] when comparing 3D printed model to 3D model of the patient and 3.14% to 2.67% [19, 20] when comparing 3D printed model to dry bone structure. The accuracy of material extrusion has been found varying from 0.22% to 1.11% [21, 22] comparing 3D printed model to 3D model of the patient. Most measurements are done manually using calipers or with coordinate measuring machine.

Some reports related to vascular anatomy are found [23] and even some commercial service related this. Still the potential of 3D printing in preoperative models in other areas has not been discovered and scientific literature is missing. The purpose of this research is to demonstrate possibilities of medical models with 3D printing in the areas that are so well know.

## 2. Materials and Methods

The process starts from medical imaging. For bony structures and for contrast agents computed tomography is current best solution. The imaging produces medical images in DICOM (Digital Imaging and Communications in Medicine) format and accordingly the image layers 3D model from imaged subject can be reconstructed. This 3D model usually requires removing unwanted geometry and errors before 3D printing. If the bony structures are not well connected to each other additional support structures are needed to hold the physical model together and these can be 3D designed before manufacturing.

### 2.1. Heart Model

**Figure 1:**
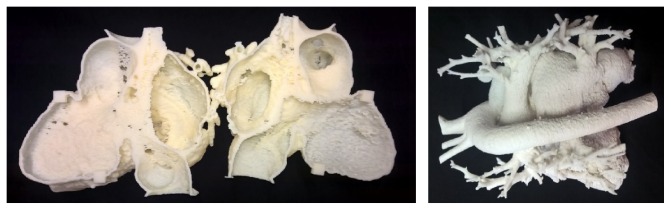
Hollow heart made by material extrusion and solid heart made by binder jetting.

Heart operation requires well-known anatomy of the heart. Normally hearts consist of similar structures; there are only small size and shape variations. In the cases where there are deformations or heart has been previously operated on there is a need for more accurate anatomic examination. Presented case patient has previous deformations and operations so surgeons feel that preoperative model would help them to plan the surgery beforehand and achieve better results. The heart was imaged with computed tomography, using contrast agent to separate heart from surrounding tissues. Layer thickness in the imaging was 600 *μ*m. 3D model was reconstructed using Osirix 5.7 (open source) with 130 Hounsfield (HU) value. For other model preparation such as repairing and hollowing 3Data Expert 10.2.1 (DeskArtes Oy) was selected as software. First the different shells were separated from each other using verified shell and repair command. In the same function also gaps thinner than 0.17 mm were stitched; fill all gaps after that, and remove tiny shell less than 0.01% of total size. The automatic repairing was performed three times and after that errors left were repaired manually one triangle per time. When the model was repaired enough it was hollowed using offset command with 2 mm offset. After offsetting model was again automatically and manually repaired. The model was separated into two parts using split command. Two different kinds of 3D model from heart were made, hollow one with 2 pieces and solid one. Both models are shown in Figure 1.

Two models of heart were 3D printed, one from inner structure with material extrusion process and one with outer structure with binder jetting process. In material extrusion Uprint SE Plus printer (Stratasys Ltd.) was used with layer thickness of 0.254 mm form ABS plus material. In binder jetting Zprinter 450 (3D Systems, Inc.) was the printing device with layer thickness of 0.1 mm and ZP151 as material. The hollow one was that which surgeons see more beneficiary since you can see inside, but also solid one gave good view about the anatomy. Biggest problem was that since heart is always pumping blood it moves during imaging and it might be that contrast agent does not flow to each desired location at the same time.

### 2.2. Ankle Models

**Figure 2:**
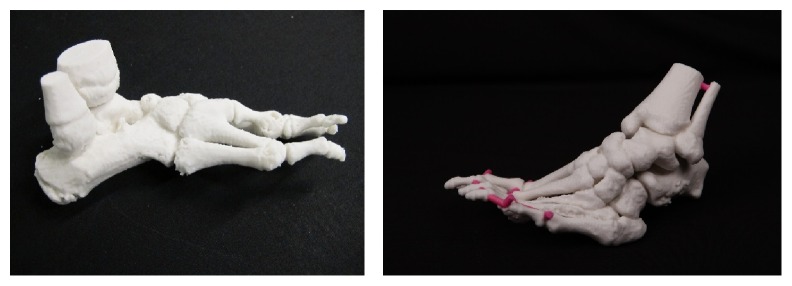
Preoperative model of ankle made by binder jetting with monochrome and with colors.

Deformation or trauma in the ankle is challenging since there is moving joint in it. It might be hard to understand how different parts of ankle move in the ankle and especially this is hard when the anatomy is abnormal. For imaging ankles, computed tomography was used with layer thickness 625 *μ*m. For repairing, hollowing, coloring, and other model preparation 3Data Expert 10.1 (DeskArtes Oy) was selected as software and Rhinoceros 4.0 was used to create geometry that connects different bones. The model was first verified and repaired with parameters stich gaps thinner than 0.17 mm; fill all gaps after that and remove tiny shell less than 0.01% of total size. The automatic repairing was performed two times and couple of errors were repaired manually. For repaired model line with dots was created in Rhinoceros and then solid pipe was created around it. Then the models were solidified to one shell using Boolean operations in 3Data Expert. Two ankles were segmented with 100 HU for both models using Osirix 5.7 (open source). One model was 3D printed with binder jetting method using Zprinter 450 (3D Systems, Inc.) in monochrome and the other with same 3D printer in color mode. For both material was ZP151 and photos of both are shown in Figure 2.

### 2.3. Models of Backbones

In scoliosis backbone is more in form of S or C than straight. In early phases, it can be treated with external support that supports backbone to grow straighter. Since deformation can be in every direction and no common form exists, every patient requires individual treatment. Planning of the treatment is quite hard with only 2D computed tomography slices or virtual model in computer screen. For 3D printing of backbones computed tomography with layer thickness 625 *μ*m was selected. HU value for the first one was 200 HU and for the second one 225 HU when using Osirix 5.7 (open source) software. For repairing, hollowing, coloring, and other model preparation 3Data Expert 10.1 was used. The models were first verified and repaired with parameters stich gaps thinner than 0.15 mm; fill all gaps after that and remove tiny shell less than 0.01% of total size. The automatic repairing was performed four times and couple of errors were repaired manually. Rhinoceros 4.0 was used adding supporting tube geometry to connect different vertebras together. Then the models were solidified to one shell using Boolean operations in 3Data Expert.

**Figure 3:**
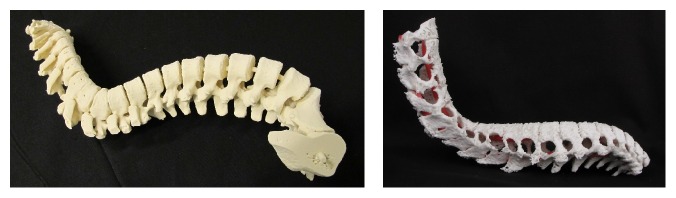
Scoliosis backbone with binder jetting without and with colors.

Since the first one was quite big binder jetting process and VX1000 (voxeljet AG) 3D printer was selected with PMMA as a material, for the second one binder jetting with Zprinter 450 (3D Systems, Inc.) was selected with ZP151 material in full color mode. Scoliosis backbone with binder jetting without and with colors is shown in Figure 3.

### 2.4. Knee Models

Similar to ankle knee is challenging to operate. There are different bones moving against each other with cartridge between them. From 2D slice images it is hard to figure out the true 3D shape of the bones. 3D printing allows seeing replicas of the bones in natural size and touching them. It is also possible to simulate moving of the joint, as imaging computed tomography was used with layer thickness 625 *μ*m. Both models were segmented using Osirix 5.7 (open source) software with 150 HU. The models were first verified and repaired using 3Data Expert 10.1 with parameters stich gaps thinner than 0.2 mm; fill all gaps after that and remove tiny shell less than 0.01% of total size. The automatic repairing was performed one time and couple of errors were repaired manually. Extra shells were removed before printing.

**Figure 4:**
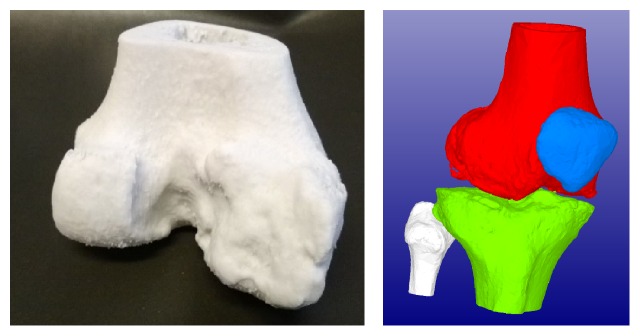
Physical and virtual preoperative model of knee.

Binder jetting with Zprinter 450 (3D Systems, Inc.) was selected as a process. The material used was ZP151. Both preoperative knee models were 3D printed in monochrome mode and in Figure 4 physical model of first one and virtual model of second one are shown.

### 2.5. Model of Pelvis

Malposition of pelvis affects whole body through backbone and legs. Traumas in this are common with older people related to fall or slip. Sometimes children may have deformation related to pelvis and usually this is related example to scoliosis. For pelvis preoperative models computed tomography with layer thickness 625 *μ*m was selected. For segmentation 250 HU was selected for the first one and 300 HU for the second one. Models were repaired, colored, and prepared using 3Data Expert 10.1 (DeskArtes Oy) which was also used to generate support geometry to hold physical model together. First the shells were separated using verified shell and extra shells removed. Repair command was run two times and selected parameters were stich gaps thinner than 0.17 mm; fill all gaps after stitching and remove tiny shell less than 0.01% of total size. After that errors left were repaired manually triangle by triangle. After repairing, support geometries were created creating cylinders to the 3D model and moving cylinders to desired locations. Multiple join operation was used to connect shell together.

**Figure 5:**
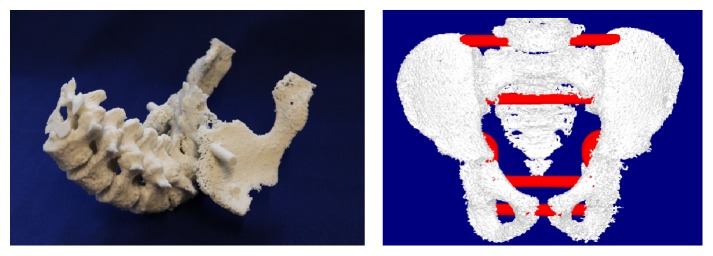
Physical and virtual preoperative model of pelvis.

Both were 3D printed using binder jetting method with Zprinter 450 (3D Systems, Inc.). The material was ZP151. Photo of the first model and screen capture of virtual model are shown in Figure 5.

### 2.6. Accuracy of the Models

All models were visually inspected after manufacturing. Two models were selected for more accurate measurements: hollow heart made by material extrusion and preoperative model of knee made by binder jetting. In the heart model inner structure of the other half and in the knee joint surface were 3D measured using ATOS Core 3D (GOM mbH, Germany). The measurements were compared against 3D model used for 3D printing with GOM Inspect V7.5 SR2 (GOM mbH, Germany).

## 3. Results and Discussion

Preoperative models are mostly needed cases where anatomy of the patient varies from normal. This occurs in deformations and with children since their body is still growing and developing and in traumas. Examples 3D printed preoperative models were created from heart, ankle, backbone, knee, and pelvis with different processes and materials. In each case understanding of anatomy was better compared to looking only at 2D slice images. The surgeons estimated that preoperative models helped them to perform surgery. Also better planning reduces the average time used for surgery.

Medical imaging with layers always loses data. When 3D model is created from medical images geometry between layers is always mathematically calculated estimation. The smaller the layer thickness in imaging the better and more accurate the 3D model created from it. With patients radiation dose leads to the fact that layer thickness cannot be decreased more. In 3D printing selected layer thickness was the smallest one available in the selected printers. The thickness is smaller than layer thickness in imaging. Layer thickness of 3D printing generates dimensional errors to the physical model but imaging can cause more errors. Also if layer thickness of 3D printing is too high stair step effect between layers can be seen and it affects the accuracy.

**Figure 6:**
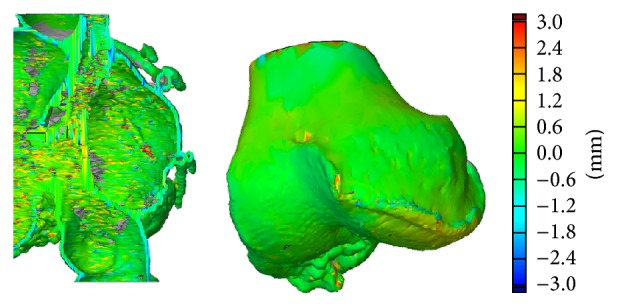
Result of 3D measurement for hollow heart model and for knee model. Scale ±3.0 mm.

In the heart model made by material extrusion overall accuracy was approximately ±1.5 mm when comparing 3D printed model to virtual 3D model. Maximum errors were approximately ±3.0 mm at thin walls, sharp corners, and small holes. Also the surface of the 3D model and the 3D print was rough because of imaging soft tissue. In the knee model made by binder jetting overall accuracy was approximately ±0.75 mm when comparing 3D printed model to virtual 3D model. Maximum errors were approximately ±2.5 mm at sharp corners and small holes. One reason for errors in small holes might be postprocessing where 3D printed part is dipped in cyanoacrylate and that can accumulate in the holes. The measurement results for both heart model and knee model are shown in Figure 6. Bone structures are more accurate in medical imaging than soft tissue structures and therefore produce better results in 3D printing. Binder jetting was found more accurate in medical models than material extrusion.

Currently preoperative models from bone applications can be easily done if the imaging quality and layer thickness are sufficient. One notice about image quality is that some hospitals remove some parts of images when archiving these for saving storage space. Therefore, images should be taken directly from imaging workspace not from archives. Next applications would be soft tissue and organ applications where more processing of medical images is needed. One possibility is to use contrast agent to increase difference in HU values between organ and tissue next to it.

Ones that would require development are models made with material jetting process and multimaterial approach. Adding both hard and soft materials to preoperative medical models it would be possible to mimic bone, ligaments, cartilage, and soft tissue in same model.

## 4. Conclusion

In future, more and more applications will be seen from other areas than dental and craniomaxillofacial ones in 3D printing of preoperative models. Surgeons should be aware of the new possibilities and in most cases help from mechanical engineering side is needed. Communication between surgeons and engineers should be developed further and research effort should be focused on preoperative medical models since the potential has not been reached yet. In future a fresh test subject should be imagined using computed tomography, 3D model created from the images, and the 3D model printed. Tissues of fresh subject should be removed and 3D bone structures measured. The measurement between original bones and 3D printed copy should be compared.
